# Lower Fractions of *TCF4* Transcripts Spanning over the CTG18.1 Trinucleotide Repeat in Human Corneal Endothelium

**DOI:** 10.3390/genes12122006

**Published:** 2021-12-17

**Authors:** Ida Maria Westin, Andreas Viberg, Berit Byström, Irina Golovleva

**Affiliations:** 1 Department of Medical Biosciences, Medical and Clinical Genetics, University of Umeå, 901 85 Umeå, Sweden; idamaria.westin@umu.se; 2 Department of Clinical Sciences, Ophthalmology, University of Umeå, 901 85 Umeå, Sweden; andreas.viberg@umu.se (A.V.); berit.a.bystrom@umu.se (B.B.)

**Keywords:** *Transcription Factor 4* (*TCF4*), Fuchs corneal dystrophy, alternative transcripts, ddPCR, mRNA expression

## Abstract

Fuchs’ endothelial corneal dystrophy (FECD) is a bilateral disease of the cornea caused by gradual loss of corneal endothelial cells. Late-onset FECD is strongly associated with the CTG18.1 trinucleotide repeat expansion in the *Transcription Factor 4* gene (*TCF4*), which forms RNA nuclear foci in corneal endothelial cells. To date, 46 RefSeq transcripts of *TCF4* are annotated by the National Center of Biotechnology information (NCBI), however the effect of the CTG18.1 expansion on expression of alternative *TCF4* transcripts is not completely understood. To investigate this, we used droplet digital PCR for quantification of *TCF4* transcripts spanning over the CTG18.1 and transcripts with transcription start sites immediately downstream of the CTG18.1. *TCF4* expression was analysed in corneal endothelium and in whole blood of FECD patients with and without CTG18.1 expansion, in non-FECD controls without CTG18.1 expansion, and in five additional control tissues. Subtle changes in transcription levels in groups of *TCF4* transcripts were detected. In corneal endothelium, we found a lower fraction of transcripts spanning over the CTG18.1 tract compared to all other tissues investigated.

## 1. Introduction

Fuchs endothelial corneal dystrophy (FECD) is a bilateral disease of the cornea caused by gradual loss of corneal endothelial cells. The corneal endothelium (CE) is a monolayer of cells lying on its basement membrane on the innermost surface of the cornea, and functions both as a barrier and as a pump, to maintain the corneal optical transparency. When the endothelial cell loss reaches a critical point, the aqueous humor inside the eye flows more freely into the cornea and results in corneal swelling and vision loss.

Early-onset FECD caused by missense mutations in *COL8A2* gene usually starts at a young age and advances to late stage in the fourth decade of life [1,2,3]. First symptoms of late-onset FECD are usually seen at mid-40 s and later, and the disease was first associated with an intronic SNP, rs613872 in *Transcription Factor 4* gene (*TCF4)* back in 2010 [4]. However later, a much stronger association was found to an expansion of cytosine-thymine-guanine (CTG)_n_ repeat, known as CTG18.1 [5], in intron 3 of the *TCF4* gene [6]. Currently, association of the *TCF4* repeat expansion (*n* > 50) and FECD has been reported in several populations [7,8,9,10,11]. In other studies, association of FECD and (CTG)_n_ repeat length over 40 has also been shown [12,13,14,15]. Functional analyses of the *TCF4* RNA transcripts spanning over the expanded CTG18.1 have revealed that the repeat expansion tract folds into secondary structures termed RNA nuclear foci in corneal endothelial cells [16,17,18].

TCF4 is a member of the class I basic-helix-loop-helix (bHLH) family of transcription factors that can bind to the DNA motif called E-box (CANNTG) and modulate transcription. These E-box regulatory sites are found in promoters and enhancer elements of numerous genes and regulate tissue specific gene expression [19]. Depending on *TCF4* transcript, the protein product may encompass different activation domains interacting with different transcriptional co-activators, which regulate transcription in a competitive manner. Besides activation domains, domains with repressing activity are also present within TCF4, which makes the entire TCF4 regulation of transcription very complex [20]. To date, 46 RefSeq transcripts of the *TCF4* gene are annotated by the National Center of Biotechnology information (NCBI), out of which 25 transcripts result from sequences that span the CTG18.1 and seven have transcription start sites (TSS) immediately at the 3′-end of the CTG18.1 repeat. In FECD, the relationship between the CTG18.1 expansion and the expression of any of the aforementioned 32 transcripts in CE is not well studied, although earlier studies of expression of total *TCF4* and a few specific transcripts showed conflicting results [7,10,16,21,22].

In this study we aimed to investigate if the *TCF4* (CTG)_n_ expansion (*n* > 40) (*TCF4*^+^), present in the majority of FECD patients affects the mRNA expression of *TCF4* transcripts spanning over the CTG18.1 or transcripts with TSS immediately at the 3′-end of the (CTG)_n_. We used digital droplet PCR for quantification of these specific *TCF4* transcripts (NCBI annotated RefSeq) in CE and in white blood cells of *TCF4*^+^ and *TCF4*^−^ (*n* < 40) FECD patients, *TCF4*^−^ non-FECD controls, and in five additional control tissues.

## 2. Materials and Methods

Biological samples. CE was obtained from 4 non-FECD corneal donors and placed in RNAlater (Invitrogen, Waltham, MA, USA). The remaining corneal tissue was placed in a separate RNAlater vial and used for genotyping. These non-FECD human corneas had been stored in nutrient medium before obtaining the CE, and they were from deceased individuals who had chosen, when alive, to donate their corneas post-mortem for research, through written consent and according to Swedish law. CE from 5 FECD patients was collected during routine Descemet Stripping Automated Endothelial Keratoplasty (DSAEK). Before the surgery, the patients received both written and oral information about the study, and written informed consent was obtained. The corneal specimen was placed in RNAlater, washed twice with phosphate buffered saline (PBS), and frozen in −80 °C on the same day as surgery. Peripheral blood was obtained from 20 patients with FECD, and the white blood cells (WBC) were used in the downstream analysis. Commercially available total RNA, extracted from single individuals with unknown *TCF4* (CTG)_n_ genotype, from human skin (Zyagen, San Diego, CA, USA), human brain (Zyagen), human skeletal muscle (Zyagen), human fetal brain (Cell Applications, San Diego, CA, USA), and human fetal skin (BioCat, Heidelberg, Germany) were used in the study as control tissues. The study was approved by the Swedish Ethical Review Authority (2019-01744) and all human tissues were handled under the guidelines based on the tenets of the Declaration of Helsinki developed by World Medical Association (2013).

DNA extraction. Genomic DNA from WBC was extracted using modified salting out protocol [23]. Genomic DNA from non-FECD donor corneas was extracted using NucleoSpin Tissue XS (Macherey-Nagel, Düren, Germany). The DNA from the corneas was eluted in 20 µL BE buffer (provided by the kit).

RNA extraction and cDNA conversion. Whole blood from FECD patients was lysed in a buffer containing 130 mM NH_3_Cl, 2 mM NH_3_HCO_3_ and 0.02% diethylpyrocarbonate (DEPC). RNA was extracted from the remaining WBC using TRIzol Reagent^TM^ (Invitrogen). RNA was extracted from the CE from FECD patients and non-FECD corneal donors with miRNeasy Micro Kit (Qiagen, Hilden, Germany).

Reverse transcription was done according to manufacturer’s instructions with SuperScript™ IV VILO™ Master Mix with ezDNase™ Enzyme (Invitrogen). For each tissue type and for each RNA extraction method, a “no reverse transcriptase” control was included to verify absence of genomic DNA contamination in subsequent analysis. Assuming linearity for RNA input and cDNA output as demonstrated by the manufacturer of the kit, all cDNAs were diluted to 1 ng/µL.

*TCF4* genotyping. To determine *TCF4* repeat length, short tandem repeat PCR (STR-PCR) was used. STR-PCR master mix contained 40 ng of genomic DNA, 0.3 µM forward primer (5′-6FAM-AAATCCAAACCGCCTTCCAA-3′), 0.3 µM reverse primer (5′-AATGCACACCTTCCCTGAGT-3′), 0.2 mM dNTP (Roche Diagnostics, Mannheim Germany), 1X PCR Buffer II (Applied Biosystems, Waltham, MA, USA), 1.5 mM MgCl2 Solution (Applied Biosystems), 10% DMSO (Sigma-Aldrich, Saint Louis, MO, USA) and 0.05 U AmpliTaq GoldTM DNA Polymerase (Applied Biosystems). PCR conditions for STR-PCR were set as followed: 95 °C for 10 min, 30 cycles of: 94 °C for 30 s, 58 °C for 30 s, and 72 °C for 30 s, and a final extension step at 72 °C for 10 min. To rule out possible large expansion in samples presenting only one peak on electropherograms, triplet repeat primed PCR (TP-PCR) was used. TP-PCR mix was made by using the same forward primer as in STR-PCR together with 0.03 µM TP-CAG primer (5′-CAGGAAACAGCTATGACCCAGCAGCAGCAGCAG-3′), 0.3 µM TP-flag primer (5′-CAGGAAACAGCTATGACC-3′), 1.25 M Betaine (Sigma-Aldrich, Saint Louis, MO, USA), and up to 200 ng genomic DNA. The PCR profile for TP-PCR was 95 °C for 10 min, 10 cycles of 95 °C for 30 s, 58 °C for 30 s (with −0.5 °C/cycle), and 72 °C for 4 min, thereafter 30 cycles of 95 °C for 45 s, 58 °C for 45 s, and 72 °C for 4 min (+15 s/cycle), and lastly an extension step at 72 °C for 10 min. PCR products from STR-PCR and TP-PCR were run on ABI3500 Dx Genetic Analyzer (Applied Biosystems) and electropherograms were processed in GeneMapper Software 5 (Applied Biosystems). Expansions detected by TP-PCR were denoted as ≥125 repeats. In this study, individuals with *TCF4* (CTG)_n_ repeats >40 are defined as *TCF4*^+^ and individuals with <40 (CTG)_n_ repeats are defined as *TCF4*^−^.

**Figure 1:**
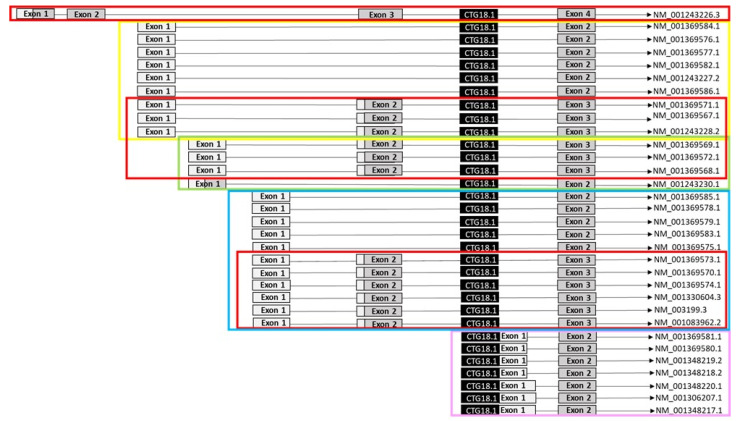
Schematic overview (not in scale) of *TCF4* transcripts targeted by five TaqMan**^®^** gene expression assays. Assay A shown in yellow targets 9 *TCF4* transcripts; assay B shown in green targets 4 *TCF4* transcripts; assay C shown in blue targets 11 *TCF4* transcripts; assay E shown in purple targets 7 *TCF4* transcripts; assay D shown in red targets 13 *TCF4* transcripts, 12 of which overlap with those targeted by assays A, B, and C. mRNAs targeted by assay A-D span over the CTG18.1 tract and mRNAs targeted by assay E have TSS immediately at the 3′-end of the (CTG)_n_ tract. Light grey boxes represent untranslated exons, dark grey boxes represent translated exons, and relative position of the CTG18.1 in the *TCF4* gene is shown in black. NM—NCBI Reference Sequence (RefSeq).

*TCF4* gene expression with digital droplet PCR (ddPCR). Five TaqMan^®^ gene expression assays (Thermo Fisher Scientific, Waltham, MA, USA) were FAM-labelled to capture all transcripts covering the *TCF4* (CTG)_n_ repeat tract in genomic sequence or having TSS in close proximity to the 3′-end of the triplet repeat (Figure 1). Alamut visual version 2.14 (Sophia Genetics, Saint Sulpice, Switzerland) and NCBI annotated RefSeq was used to manually control alignment of TaqMan assays to *TCF4* transcripts. All assays were coded for easy handling; A, B, C, D, and E, and together they targeted 32 transcripts (Figure 1).

All FAM-labelled assays were custom designed except for assay D (Hs00971339_m1, Thermo Fisher) (Table 1).

For internal comparison and for total *TCF4* expression levels, a TaqMan^®^ gene expression assay labelled with VIC (Hs00162613_m1), targeting all 46 *TCF4* NCBI RefSeq transcripts, was added to each reaction. In short, each coded ddPCR reactions (A–E) were run separately in 20 µL reactions with 1x ddPCR Supermix for Probes (no dUTP) (Bio-Rad, Hercules, CA, USA), 1x TaqMan probe (FAM), 1x TaqMan probe (VIC), and 1 ng cDNA. For droplet generation, Droplet Generation Oil for probes (Bio-Rad) and QX200 Droplet Generator (Bio-Rad) was used. PCR program for droplet reactions was for A, B, D, and E assays as followed; initial denature step at 95 °C for 10 min, 40 cycles of; 94 °C for 30 s and 60 °C for 1 min (ramp rate −2 °C/s), ending with 98 °C for 10 min and 4 °C infinity. Assay C was run with 50 cycles, annealing at 60 °C for 30 s (ramp rate −2 °C/s), and an extra elongation step at 72 °C for 30 s (ramp rate −2 °C/s). QX200 Droplet Reader (Bio-Rad) was used for droplet detection and Absolute Quantification (ABS) was used as detection method in QuantaSoft software version 1.7.4.0917 (Bio-Rad, Hercules, CA, USA). All quantifications were presented as copies/µL by the software and the ratio of each coded transcripts versus total *TCF4* expression was calculated for each sample. For all assays, “no reverse transcriptase” controls were run to rule out genomic DNA contamination.

Statistical Analysis and data plotting. Gene expression was plotted with the open sourced softwares Jupyter Notebook version 6.0.0 (https://jupyter.org), Python version 3.7.3 (https://www.python.org), Pandas version 0.24.2 (https://pandas.pydata.org), Matplotlib version 3.4.2 (https://matplotlib.org) and Numpy version 1.17.0 (https://numpy.org). Two-sided Mann-Whitney U was calculated with Scipy version 1.5.4 (https://scipy.org) to detect significant differences in gene expression between groups. Differences with a *p* value less than 0.05 were considered statistically significant. For the comparison of gene expression between CE and all other tissues, the mean of each assay for each sample group was used in the calculation, and not individual values.

Inter quartile range (IQR) was used to measure the variability of *TCF4* (CTG)_n_ repeat lengths within groups.

## 3. Results

### 3.1. FECD Patients and Controls Characteristics

Of the CE samples, 3 out of 4 non-FECD corneal donors were females compared to 1 out of 5 FECD patients (Table 2). The mean age of non-FECD corneal donors was 59 ± 21 years (mean ± SD). The donated corneas had been stored in nutrient medium for 45 ± 20 days (mean ± SD) before nucleic acid extraction. In comparison, the mean age of the FECD patients was 76 ± 8 years (mean ± SD) at time for the DSAEK.

The mean age of FECD patients who donated WBC was 73 ± 9 years (mean ± SD) for *TCF4*^+^ cases and 74 ± 3 years (mean ± SD) for *TCF4*^−^ cases.

### 3.2. TCF4 Genotyping

None of the non-FECD corneal donors (*n* = 4) had *TCF4* (CTG)_n_ expansion and were therefore termed *TCF4*^−^. The median *TCF4* repeat length of the longest allele in this group was 18 repeats (IQR = 3.4) (Table 2).

All FECD patients undergoing DSAEK (*n* = 5) had (CTG)_n_ repeat lengths >40 and were denoted as *TCF4*^+^. The median *TCF4* repeat length of the longest allele among these cases was 87 repeats (IQR = 26) (Table 2).

Whole blood was available from two groups of FECD patients, one (*n* = 16) with *TCF4* (CTG)_n_ repeat length over 40 (median 94 repeats, IQR = 15.5), (*TCF4*^+^), and another group (*n* = 4) with *TCF4* (CTG)_n_ length less than 40 (median 14 repeats, IQR = 4), (*TCF4*^−^) (Table 2).

### 3.3. TCF4 mRNA Expression by Digital Droplet PCR (ddPCR)

In this study, all *TCF4* transcripts (NCBI annotated RefSeq) (*n* = 32) spanning over the CTG18.1 repeat and transcripts starting immediately at the 3′ end of the repeat were quantified with ddPCR. Transcripts were divided into groups (A to E) depending upon which assays targeted them (Figure 1). All assays were normalized against total mRNA *TCF4* expression to detect quantitative alterations in specific transcript groups.

**Figure 2:**
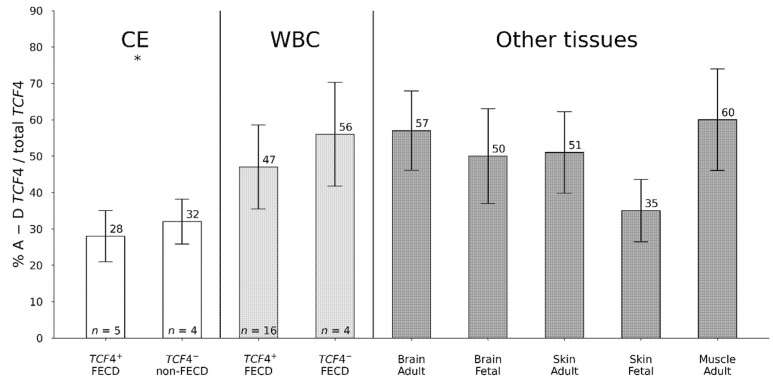
Each bar displays a percentage of a ratio of sum of mRNA expression for TaqMan^TM^ assay A, B, C, and D to total *TCF4* gene expression. These assays detect only *TCF4* transcripts that span over the CTG18.1 tract. Numbers on top of bars indicate % of total *TCF4* expression. Numbers at bottom of bars indicate number of individuals in each group. *TCF4*^+^—individuals harbouring *TCF4* (CTG)_n_ with more than 40 repeats (*n* > 40), *TCF4*^−^—individuals harbouring *TCF4* (CTG)_n_ with less than 40 repeats (*n* < 40). CE-corneal endothelium, WBC-white blood cells, * *p*-value = 0.04 in comparison of CE A–D *TCF4* expression (*n* = 9) to all other tissues (*n* = 25) presented in the Figure.

CE (*n* = 9) had the lowest fraction of transcripts spanning over the *TCF4* (CTG)_n_ repeat expansion regardless of FECD/*TCF4* status compared to WBC and all other tissues analysed (*n* = 25) (*p* = 0.04) (Figure 2). Moreover, *TCF4*^+^ CE from FECD patients had the lowermost fractions of these transcripts, with ~4% lesser than CE from *TCF4*^−^ non-FECD donors (Figure 2), although the difference was not significant.

### 3.4. Differential TCF4 mRNA Expression in Corneal Endothelium

**Figure 3:**
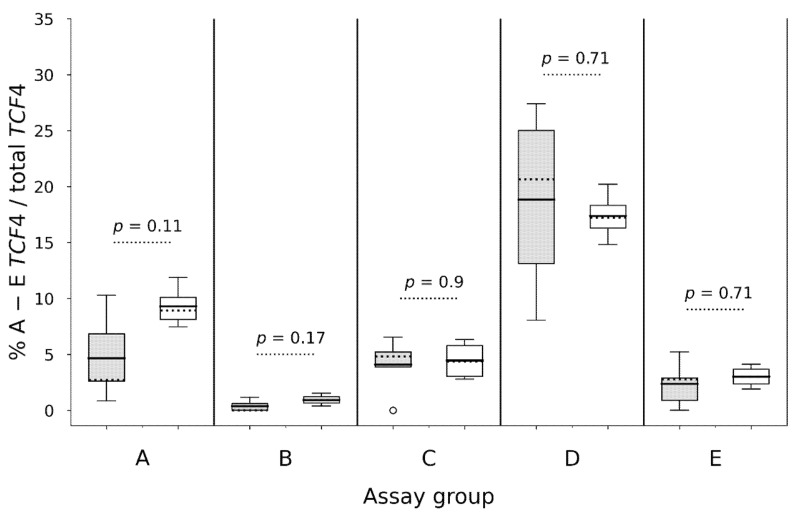
mRNA gene expression by TaqMan^TM^ assays (**A**) to (**E**) targeting *TCF4* transcripts (*n* = 32) spanning over the (CTG)_n_ repeat (**A**–**D**) and transcripts starting immediately at the 3′ end of the repeat (**E**) in FECD *TCF4*^+^ CE (grey) and non-FECD *TCF4*^−^ CE (white). Within boxes, dotted lines display medians and continuous lines display means. Empty circles display outliers.

*TCF4* mRNA expression in CE from *TCF4*^+^ FECD patients was compared with gene expression in CE from *TCF4*^−^ non-FECD donors by using specific assays A to E. Assay A and D targeted nine and 13 transcripts, respectively, and had three overlapping target transcripts (Figure 1). These two assays demonstrated a noticeable variability in gene expression in the *TCF4*^+^ FECD group, which was not seen among the *TCF4*^−^ non-FECD donors, though the differences were not statistically significant (Figure 3). Fraction of transcripts detected by assay A also displayed a trend towards higher expression in the CE from *TCF4*^−^ non-FECD donors (~10%) than in the CE from *TCF4*^+^ FECD patients (~5%) (*p* = 0.11) (Figure 3). Moreover, transcripts detected by assay D had the highest fraction in the CE of all the assays (A to E) (Figure 3) analyzed.

Assays B, C, and E did not reveal any difference in *TCF4* expression in the CE between these two groups, although transcripts detected by assay E displayed some variability in the *TCF4*^+^ FECD group.

### 3.5. Comparison of TCF4 mRNA Expression in Individual Assays (A to E) in WBC and CE

Next, using individual assays, we focused our analysis on WBC, and examined differential *TCF4* mRNA expression in WBC of *TCF4*^+^ and *TCF4*^−^ FECD patients and also we compared gene expression in CE and WBC of *TCF4*^+^ FECD patients.

**Figure 4:**
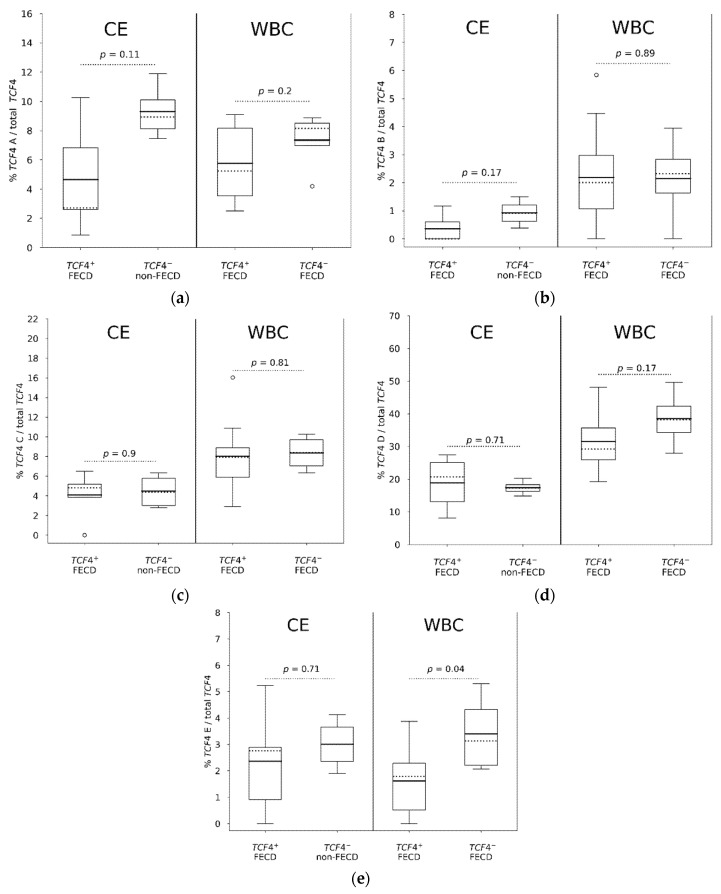
mRNA gene expression probed by TaqMan^TM^ assays A to E in corneal endothelium and WBC targeting *TCF4* transcripts spanning over the CTG18.1 (**a**–**d**) and *TCF4* transcripts starting immediately at the 3′ end of the (CTG)_n_ repeat tract (**e**). Within boxes, dotted lines display medians and continuous lines display means. Empty circles display outliers. *TCF4*^+^—individuals harbouring *TCF4* (CTG)_n_ with more than 40 repeats (*n* > 40), *TCF4*^−^—individuals harbouring *TCF4* (CTG)_n_ with less than 40 repeats (*n* < 40). CE—corneal endothelium, WBC—white blood cells.

Assay A targeting nine *TCF4* transcripts (Figure 1) had similar diverse expression level in WBC from *TCF4*^+^ FECD patients as in CE from *TCF4*^+^ FECD patients (Figure 4a) without statistical difference between these two groups (*p* = 0.3). Also, there was no statistical difference in the fraction of transcripts targeted by assay A in WBC between *TCF4*^+^ and *TCF4*^−^ FECD patients (*p* = 0.2) (Figure 4a).

Assay B targeted four *TCF4* transcripts (Figure 1) and the fraction of transcripts detected by this assay was higher in WBC than in the CE in general, although the levels were still relatively low (~2%) (Figure 4b). There was no significant difference between WBC from *TCF4*^+^ FECD patients and WBC from *TCF4*^−^ FECD patients (*p* = 0.89) but the higher expression level in WBC resulted in a statistical difference between CE from *TCF4*^+^ FECD patients and WBC from *TCF4*^+^ FECD patients (*p* = 0.014). Overall, the fraction of transcripts detected by assay B were the lowest among all assays (Figure 4b).

Assay C targeting 11 *TCF4* transcripts (Figure 1) showed higher fraction of transcripts detected by this assay in WBC than in CE from *TCF4*^+^ FECD patients (*p* = 0.014) (Figure 4c). No statistical difference in gene expression was found in WBC between *TCF4*^+^ and *TCF4*^−^ FECD patients (*p =* 0.81) (Figure 4c).

Assay D targeting 13 *TCF4* transcripts (Figure 1) revealed a higher fraction of transcripts detected by this assay in WBC than in the CE in general (Figure 4d). Statistical difference was observed between CE and WBC from *TCF4*^+^ FECD patients (*p* = 0.018). In WBC, there was no difference in gene expression between *TCF4*^+^ and *TCF4*^−^ FECD patients (*p* = 0.17).

Expression of seven *TCF4* transcripts with TSS immediately downstream the (CTG)_n_ repeat interrogated by assay E (Figure 1) demonstrated lower gene expression in WBC of *TCF4*^+^ FECD patients than in WBC from *TCF4*^−^ FECD (*p =* 0.04) (Figure 4e, Supplementary Figure S1). A similar trend was seen in CE between *TCF4*^+^ FECD patients and *TCF4*^−^ non-FECD controls and no difference in gene expression was seen between WBC and CE from *TCF4*^+^ FECD patients (*p* = 0.56).

## 4. Discussion

In this study, we investigated if the *TCF4* (CTG)_n_ expansion (*n* > 40) present in the majority of FECD patients affects the mRNA expression of *TCF4* transcripts spanning over the CTG18.1 repeat or transcripts with TSS immediately at the 3′-end of the (CTG)_n_. We hypothesized that an expansion >40 repeats would change expression levels of specific *TCF4* mRNA transcripts.

Due to the high homology and diversity of *TCF4* transcripts, we divided the transcripts into assay groups of A to E. Assay C and assay D targeted i.a. NM_001083962.2 (*TCF4*-B+), the canonical transcript according to NCBI and Ensembl and NM_003199.3 (*TCF4*-B−), the canonical transcript in agreement with the Universal Protein Resource Knowledgebase (UniProtKB). Transcripts detected by Assay D had the highest fractions in both WBC and CE, however assay C did not show the same level of expression. Moreover, assay D had the largest divergence among all assays in CE from FECD patients, while assay C did not. This indicates that the divergence seen in assay D must be from any other transcripts not targeted by assay C, and not from the canonical ones. Additionally, the higher fraction of transcripts detected by assay D most likely represent a sum of all transcripts, since this assay targets the most transcripts in numbers.

Transcripts NM_001369569.1, NM_001369572.1, NM_001369568.1 and NM_001369571.1, NM_001369567.1, NM_001243228.2 targeted by assay D are also targeted by assay B and assay A, respectively. Only assay A displayed similar divergence in gene expression in CE from FECD patients as assay D. This indicates that the transcripts NM_001369571.1, NM_001369567.1, or NM_001243228.2 may be the source for this divergence, although we cannot rule out the contribution of other transcripts targeted by assay A and assay D. In addition, fractions of transcripts detected by assay A had a lower proportion in CE from *TCF4*^+^ FECD patients compared to CE from *TCF4^−^* non-FECD donors, though the difference did not reach the significant threshold (*p* = 0.11). The variations in gene expression among *TCF4*^+^ FECD patients seen in assay A and assay D may mirror an effect from possible RNA foci formation, however RNA foci were not analyzed nor quantified in this study, and therefore such conclusion is merely speculative.

In *TCF4*^+^ WBC, we found statistically significant lower fraction of transcripts with TSS immediately downstream of the CTG18.1, while the expression was variable in *TCF4*^+^ CE, with a trend towards lower fraction. Lower expression of these specific transcripts has been reported previously, where reduced expression in CE from FECD patients correlated with the length of the expanded CTG18.1 [22]. In our study, the median repeat length was longer, and more samples were available for *TCF4*^+^ WBC than for *TCF4*^+^ CE, which might explain why *TCF4*^+^ CE did not reveal a significant difference.

It is worth mentioning that in this study we observed, regardless of (CTG)_n_ expansion, lower fraction of transcripts spanning over the CTG18.1 in CE, compared to WBC and other analyzed tissues. Considering the scarce total *TCF4* relative expression in the CE (~1%) [7,16,21], this lower fraction may render the CE more sensitive to any change in availability of transcripts spanning over the CTG18.1, possibly exerted by RNA foci.

*TCF4*^+^ CE had the lowermost fraction of transcripts spanning over the CTG18.1. This lower fraction can be a consequence of three scenarios: 1) either total *TCF4* expression is increased in the *TCF4*^+^ CE due to higher expression of transcripts with TSS downstream of CTG18.1 as reported by Timmusk et al. [22] and Okumura et al. [10], or 2) the lower fraction is due to lower expression of transcripts spanning over the CTG18.1, supported by results from Foja et al. [7], with sustained total *TCF4* expression as reported by Mootha et al. [16] and Ołdak et al. [21]. The third scenario is the different handling procedures of the CE material, fresh versus stored in nutrition medium prior to RNA extraction, which may affect the *TCF4* expression in the CE from the *TCF4*^−^ corneal donors, with a possibility of either more rapid degradation or lower expression of transcripts spanning over the CTG18.1 due to non-innate incubation. It is unclear how long the donated corneas were stored as non-frozen in previous stuies [7,10,16,21], as this was not reported, although Foja et al. [7] mentioned that the donated corneas had been stored in cultivation medium prior to RNA extraction. It is known that corneas stored up to seven weeks in culture medium are equally suitable for DSAEK as corneas stored less than four weeks [24], and can therefore be considered viable with no severe malfunction, while storage beyond 7 weeks is still unknown.

One drawback of this study is the small sample sizes of CE from healthy and FECD individuals (4 versus 5), which makes the statistical power less reliable when changes are subtle. Moreover, gene expression studies of *TCF4* in FECD are challenging due to the abundance and sequence similarity of known transcripts (*n* = 46), the limited number of cells attained from DSAEK method, and the limited access to surgical material.

## 5. Conclusions

In conclusion, subtle changes in transcription in groups of *TCF4* transcripts were found in CE of *TCF4*^+^ FECD patients.

Notably, regardless of (CTG)_n_ expansion, a lower fraction of transcripts spanning over the *TCF4* CTG18.1 was detected in corneal endothelium compared to brain, skin, muscle, and lymphocytes from peripheral blood. This lower fraction might contribute to FECD pathophysiology, as the CE may be more vulnerable to any change in the availability of CTG18.1 transcripts, perhaps utilized by RNA foci.

## Figures and Tables

**Table 1:** Primer and probe sequences for custom TaqMan^®^ assays.

Custom TaqMan^®^ Assay	Primer and Probe (5′ ⟶ 3′)
A	F ^1^: GTCTCTCTTTTTAAAGTCTCTTTCCTTGGAA R ^2^: TTGAGCCAGTAAAATGTCCA Probe: TGTGGCCATTTAAGATGTT
B	F: GGGATGTAAACTCGAATAAATTTCAAAGTG R: TTGAGCCAGTAAAATGTCCA Probe: AGGCTTCAGATTGTAACTGAC
C	F: TGAACGCCGCCTCGG R: TTGAGCCAGTAAAATGTCCAC Probe: TGCACGGAGAGCCC
E	F: CCATTCGTTCCTTTGCTTTTTGCA R: CCCCAGGACCCTGAGCTA Probe: TTGAGCCAGTAAAATGTC

^1^ F-forward primer, ^2^ R-reverse primer.

**Table 2:** FECD patients and controls characteristics.

	Tissue	Group	Sex	Age (Years)	*TCF4* Allele 1	*TCF4* Allele 2	*TCF4* Genotype
KFUH9	CE ^1^	control	female	78	12	25	*TCF4* ^−^
KFUH12	CE	control	female	25	12	18	*TCF4* ^−^
KFUH13	CE	control	female	56	12	18	*TCF4* ^−^
KFUH15	CE	control	male	76	12	18	*TCF4* ^−^
FUH18	CE	FECD	male	76	76	87	*TCF4^+^*
FUH19	CE	FECD	male	85	22	75	*TCF4^+^*
FUH20	CE	FECD	male	85	12	93	*TCF4^+^*
FUH21	CE	FECD	male	71	12	71	*TCF4^+^*
FUH22	CE	FECD	female	63	12	105	*TCF4^+^*
FUB1	WBC ^2^	FECD	female	62	12	95	*TCF4^+^*
FUB2	WBC	FECD	female	81	15	93	*TCF4^+^*
FUB3	WBC	FECD	female	69	81	>81	*TCF4^+^*
FUB4	WBC	FECD	male	59	17	>125	*TCF4^+^*
FUB5	WBC	FECD	female	76	37	97	*TCF4^+^*
FUB7	WBC	FECD	female	84	20	52	*TCF4^+^*
FUB8	WBC	FECD	male	75	12	95	*TCF4^+^*
FUB9	WBC	FECD	female	73	19	85	*TCF4^+^*
FUB10	WBC	FECD	male	62	27	78	*TCF4^+^*
FUB11	WBC	FECD	female	72	27	91	*TCF4^+^*
FUB12	WBC	FECD	female	84	12	67	*TCF4^+^*
FUB13	WBC	FECD	female	84	18	96	*TCF4^+^*
FUB14	WBC	FECD	female	76	19	83	*TCF4^+^*
FUB15	WBC	FECD	male	79	17	103	*TCF4^+^*
FUB16	WBC	FECD	female	59	19	98	*TCF4^+^*
FUB17	WBC	FECD	female	65	29	101	*TCF4^+^*
7753	WBC	FECD	male	70	13	13	*TCF4* ^−^
8360	WBC	FECD	female	75	15	19	*TCF4* ^−^
1494	WBC	FECD	female	78	12	15	*TCF4* ^−^
9309	WBC	FECD	male	74	13	13	*TCF4* ^−^

^1^ CE, corneal endothelium; ^2^ WBC, white blood cells.

## Data Availability

The authors declare that all data supporting the findings of this study are available within the article. The datasets generated, used, and analyzed during this study are available from the corresponding author on reasonable request.

## Supplementary Materials

The following are available online at https://www.mdpi.com/article/10.3390/genes12122006/s1, Figure S1: mRNA gene expression of *TCF4* transcripts spanning the (CTG)_n_ repeat in WBC.
