# Supplementary material for: Lower Fractions of TCF4 Transcripts Spanning over the CTG18.1 Trinucleotide Repeat in Human Corneal Endothelium
Source: Genes (Basel). 2021 Dec 17;12(12):2006. doi: 10.3390/genes12122006 (PMC8702116; doi:10.3390/genes12122006)
Supplement: Supplementary file 1 [file genes-12-02006-s001.zip › genes-1469066-supplementary.pdf]

Supplementary Figure S1.

mRNA gene expression of *TCF4* transcripts spanning the $(CTG)_n$ repeat in WBC

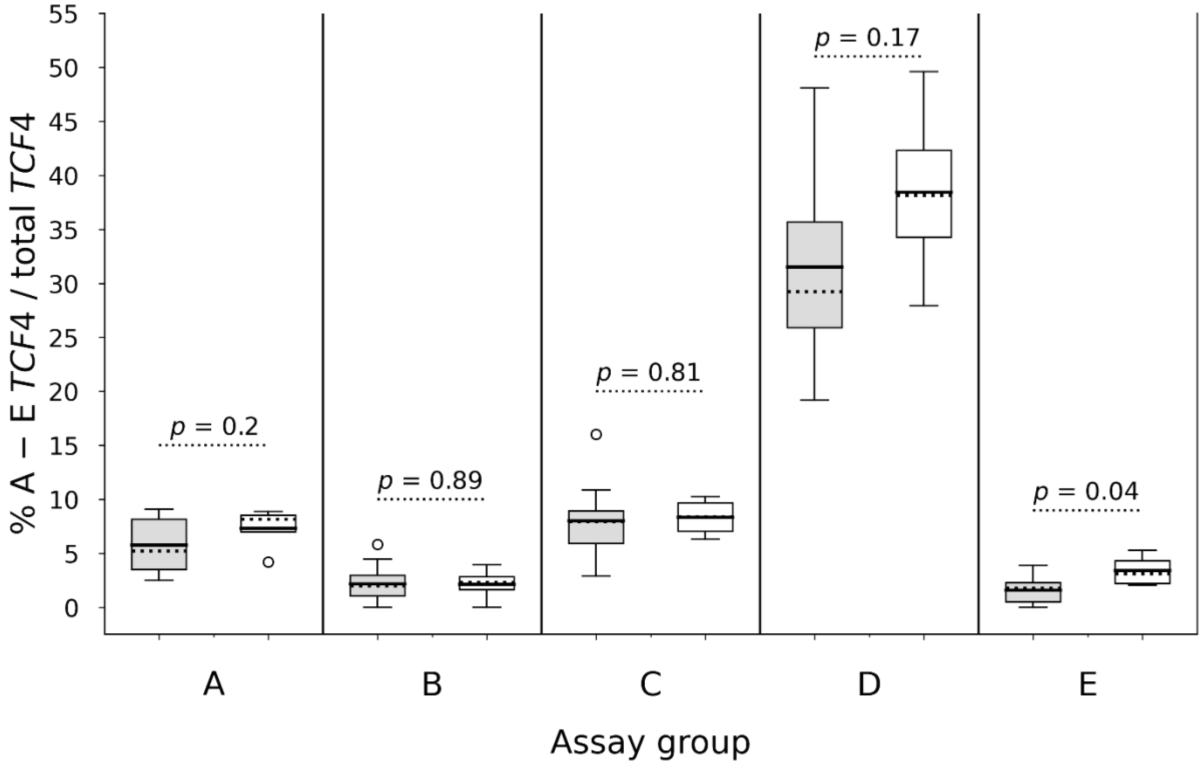

Figure S1. mRNA gene expression by TaqMan™ assays A to E targeting *TCF4* transcripts (n=32) spanning the $(CTG)_n$ repeat (A to D) and transcripts starting immediately at the 3′ end of the triplet repeat (E) in FECD *TCF4*$^+$ WBC (grey) and FECD *TCF4*$^-$ WBC (white). Within boxes, dotted lines display medians and continuous lines display means. Empty circles display outliers
